# Elevated Amniotic Fluid 8-Iso-Prostaglandin F_2_α Reveals Intrauterine Oxidative Stress in Fetuses with Congenital Heart Disease: A Prospective Case–Control Study

**DOI:** 10.3390/antiox15050586

**Published:** 2026-05-06

**Authors:** Miguel Arráez, Marta Camprubí-Camprubí, María C. Escobar-Diaz, Laura Guirado, Laura Nogué, Mar Bennasar, Narcís Masoller, Fàtima Crispi, María Dolores Gómez-Roig, Olga Gómez, Míriam Pérez-Cruz

**Affiliations:** 1 BCNatal Fetal Medicine Research Center (Hospital Clínic and Hospital Sant Joan de Déu), 08950 Barcelona, Spain; miguel.arraez@sjd.es (M.A.); laura.guirado@sjd.es (L.G.); nogue@clinic.cat (L.N.); bennasar@clinic.cat (M.B.); masoller@clinic.cat (N.M.); fcrispi@clinic.cat (F.C.); lola.gomezroig@sjd.es (M.D.G.-R.); ogomez@clinic.cat (O.G.); miriam.perez@sjd.es (M.P.-C.); 2 Spanish Network in Maternal, Neonatal, Child and Developmental Health Research, RICORS-SAMID, RD24/0013/0004, Instituto de Salud Carlos III, 28040 Madrid, Spain; 3 Neonatology Department, Sant Joan de Déu Hospital, 08950 Barcelona, Spain; 4 Cardiovascular Research Group, Sant Joan de Deu Research Institute, 08950 Barcelona, Spain; 5 Institut de Recerca Sant Joan de Déu (IRSJD), 08950 Barcelona, Spain; mariaclara.escobar@sjd.es; 6 Cardiology Department, Sant Joan de Déu Hospital, 08950 Barcelona, Spain; 7 Institut d’Investigacions Biomèdiques August Pi i Sunyer (IDIBAPS), 08036 Barcelona, Spain; 8 Centre for Biomedical Research on Rare Diseases (CIBER-ER), 28029 Madrid, Spain

**Keywords:** amniotic fluid, congenital heart disease, oxidative stress, 8-iso-prostaglandin F_2_α, F_2_-isoprostanes, fetal biomarkers, lipid peroxidation, prenatal diagnosis

## Abstract

Advances in prenatal diagnosis have improved the perinatal management of congenital heart disease (CHD). However postnatal comorbidities still persist due to multifactorial causes, which limits prenatal prediction of individual outcomes. Oxidative stress (OS), particularly lipid peroxidation, has been suggested to play a role in the development and progression of CHD, with 8-iso-prostaglandin F_2_α (8-iso-PGF_2_α) serving as a biomarker of oxidative injury. This prospective case–control study aimed to evaluate OS in fetuses with isolated major CHD by comparing amniotic fluid (AF) 8-iso-PGF_2_α concentrations with controls. A total of 123 fetuses (83 CHD, 40 controls) were included at a tertiary CHD referral center. CHD cases were subclassified according to anatomical type and expected fetal brain perfusion under placental circulation. Controls were gestational age-matched pregnancies undergoing amniocentesis for indications unlikely to affect OS. All pregnant women underwent standardized fetal biometry, Doppler assessment, and detailed echocardiography. AF samples were obtained by amniocentesis and analyzed for free 8-iso-PGF_2_α using a competitive ELISA, with values normalized to creatinine. Clinical, obstetric, and Doppler characteristics were comparable between groups. CHD fetuses showed significantly higher AF 8-iso-PGF_2_α concentrations than controls (2849 ± 1377 vs. 2088 ± 1087 ng/mg Cr, *p* = < 0.001), and remained significant after adjustment for GA, smoking status, diabetes and maternal age and body mass index (BMI). No consistent differences were observed across anatomical or hemodynamic CHD subgroups. These findings provide the first intrauterine evidence of increased lipid peroxidation in fetuses with CHD as reflected by elevated amniotic fluid 8-iso-PGF_2_α concentrations.

## 1. Introduction

Advances in prenatal diagnosis have substantially improved the clinical management of congenital heart disease (CHD), leading to increased survival and reduced early postnatal morbidity [1]. However, prenatal counseling regarding long-term prognosis and potential morbidities, including neurodevelopmental impairment, remains limited. Current risk stratification relies on CHD anatomy and hemodynamic parameters which lack sufficient precision to predict individual outcomes. This limitation has prompted interest in identifying fetal biomarkers to enhance prognostic assessment and better understand CHD pathophysiology in the context of feto-placental circulation.

Oxidative stress (OS), defined as an imbalance between the production of reactive oxygen species and antioxidant defense mechanisms, has emerged as a key contributor to the development and progression of CHD [2,3,4,5]. Experimental and clinical data suggest that sustained OS during fetal life may interfere with cardiac morphogenesis and placental function, thereby impairing fetal perfusion and brain maturation [6]. F_2_-isoprostanes are formed by the non-enzymatic peroxidation of arachidonic acid, with 8-iso-prostaglandin F_2_α (8-iso-PGF_2_α) recognized as one of the best markers of in vivo lipid peroxidation and oxidative injury [7,8,9,10,11,12], which can be accurately quantified in plasma, urine, or cerebrospinal fluid [13].

OS has been proposed to play a role both in the development of CHD and in its impact on cardiovascular function, potentially contributing to associated morbidity. To date, most evidence comes from postnatal and perioperative studies, which have shown that elevated OS, particularly in cyanotic CHD, are associated with ventricular dysfunction, prolonged mechanical ventilation, and neurological complications [11,13,14,15,16,17,18]. Increased urinary and serum 8-iso-PGF_2_α levels after cardiac surgery, together with delayed biomarker clearance, have been correlated with poorer postoperative recovery and adverse neurodevelopmental outcomes [16,18]. Consistently, a recent meta-analysis reported higher OS levels in cyanotic CHD compared with other CHD types, highlighting its potential as a biomarker of disease severity [2].

Despite this substantial postnatal evidence, data on OS during fetal life are scarce, and its role in the intrauterine environment of CHD is largely unexplored. Preliminary studies using alternative oxidative markers, such as ortho-tyrosine (o-Tyr), suggest increased oxidative activity in the amniotic fluid (AF) of fetuses with CHD, with possible associations between CHD severity and impaired brain growth [19]. However, to date, no prenatal evaluation has evaluated lipid peroxidation biomarkers, particularly 8-iso-PGF_2_α, across different CHD types.

Notably, 8-iso-PGF_2_α has also been proposed to evaluate OS in several pregnancy-related disorders involving inflammatory and/or vascular processes such as preeclampsia, fetal growth restriction, gestational diabetes, and premature rupture of fetal membranes [9,19,20,21,22,23,24,25,26,27,28]. In these conditions, elevated AF 8-iso-PGF_2_α levels have been associated with placental dysfunction and impaired fetal growth [27,28]. Recent advances in liquid chromatography–tandem mass spectrometry (LC-MS/MS) techniques now allow precise and reproducible quantification of OS biomarkers, positioning AF as a promising biological matrix to explore fetal redox homeostasis in placental-related complications and CHD, as a potential novel prognostic tool.

This study aims to (1) evaluate whether fetuses with isolated CHD exhibit increased OS in utero, as reflected by higher 8-iso-PGF_2_α concentrations in AF, compared to fetuses without major cardiac defects; and, secondarily, to (2) explore associations between AF 8-iso-PGF_2_α levels and CHD anatomical subtypes, fetal hemodynamics, and neonatal risk categories. We hypothesize that altered fetal perfusion and oxygenation in CHD create a pro-oxidant intrauterine environment that is detectable through this reliable marker of lipid peroxidation. This exploratory study seeks to generate preliminary evidence and establish the basis of future prospective research integrating biochemical, hemodynamic, and neurodevelopmental outcomes.

## 2. Materials and Methods

### 2.1. Study Design and Participants

This prospective case–control study was performed at a tertiary referral center for CHD (BCNatal, Fetal Medicine Research Center, Hospital Clínic and Hospital Sant Joan de Déu, University of Barcelona, Spain) and included pregnant women between May 2020 and December 2023. Cases comprised fetuses diagnosed with isolated major CHD whose parents consented to amniocentesis for genetic study. The control group consisted of pregnant women undergoing amniocentesis for clinical indications not expected to influence OS biomarkers. This indication included high-risk screening for aneuploidy with normal molecular karyotype, minor cardiac anomalies without hemodynamic significance, fetal cardiac anomalies not confirmed postnatally, and isolated extracardiac structural anomalies unlikely to influence AF composition. Control cases were matched to the CHD group by gestational age (GA) at amniocentesis. Exclusion criteria included the presence of genetic abnormalities and major structural anomalies known or expected to alter OS or the AF compartment, such as congenital diaphragmatic hernia, hydrops fetalis, neural tube defects and renal anomalies.

Maternal characteristics, as well as perinatal and cardiovascular outcome data, were recorded in all participants. Both cases and controls underwent ultrasonographic examinations at the time of amniocentesis and again at 32–34 weeks of gestation, using a GE Voluson E8 Expert (GE Healthcare, London, UK). Each examination included the estimation of fetal weight, standard feto-placental Doppler evaluation, and comprehensive fetal echocardiography. GA was calculated based on crown-rump length at first-trimester ultrasound [29], and estimated fetal and birth weight centiles were calculated using local reference curves [30]. Feto-placental Doppler evaluation included measurements of the pulsatility index (PI) for the uterine, umbilical, middle cerebral artery and ductus venosus. The cerebroplacental ratio was calculated by dividing the middle cerebral artery PI by the umbilical artery PI [31].

The study protocol was approved by the institutional ethics committee (PIC-124-19) and written informed consent was obtained from all participants.

### 2.2. CHD Subgroup Classification

To explore the relationship between OS and CHD, cases were subclassified according to anatomical type and expected fetal brain perfusion under placental circulation. Anatomically, fetuses were categorized as right CHD, left CHD, septal CHD, conotruncal CHD, or other types [1]. Fetal brain perfusion was classified according to the expected contribution of well-oxygenated placental blood as low, intermediate, and near-to-normal perfusion, based on previously described classifications [6,32].

The first group, representing low placental contribution, included CHD in which the fetal brain receives a low proportion of placental oxygenated blood. This category comprised mainly severe left ventricular outflow tract obstruction with reversed flow at the aortic isthmus and d-transposition of the great arteries, in which the aorta arises from the right ventricle. The second group, corresponding to intermediate placental contribution, included CHD with mixed brain oxygenation from placental and systemic blood due to abnormal intracardiac shunting. This group encompassed ventricular septal defects and conotruncal anomalies, with the exception of d-transposition of great arteries. The last group, representing near-to-normal placental contribution, mainly comprised right CHD, congenitally corrected transposition of great arteries without additional cardiac defects and those cases not meeting the criteria for the previous groups, such as coarctation of the aorta with anterograde flow in the aortic isthmus.

Additional stratification was performed based on abnormal fetal Doppler patterns, altered intracardiac shunting, and high-risk neonatal outcomes. Doppler abnormalities included obstructed aortic flow, reversed flow in the aortic isthmus, and reversed flow in the ductus arteriosus. Neonatal outcomes were classified as critical CHD, defined by the need for immediate postnatal intervention and/or prostaglandin therapy, and univentricular CHD, defined by single-ventricle physiology.

### 2.3. Sample Collection and Biomarker Measurement

AF samples were obtained via ultrasound-guided amniocentesis performed by a fetal medicine specialist, following standardized methodology [33]. A mean AF volume of 20 mL was collected per procedure, and 2 mL aliquots were stored at −80 °C until analysis.

AF 8-iso-PGF_2_α concentrations were measured using a commercially available competitive enzyme-linked immunosorbent assay (ELISA) kit (Cayman Chemical, Ann Arbor, MI, USA; item no. 516351), following the manufacturer’s instructions. This assay has been previously validated for the quantification of 8-iso-PGF_2_α in biological fluids and is widely used in clinical and translational studies assessing oxidative stress. Analytical performance characteristics provided by the manufacturer include a limit of detection (LOD) of approximately 2 pg/mL and a quantification range of 8–500 pg/mL. Intra-assay and inter-assay coefficients of variation are reported to be <10% and <15%, respectively. To ensure reliability in the AF matrix, all samples were analyzed in duplicate, and measurements were accepted only if the coefficient of variation between duplicates was <15%.

Biomarker concentrations were normalized to AF creatinine and expressed as ng/mg creatinine (ng/mg Cr).

### 2.4. Statistical Analysis

Data were analyzed using SPSS Statistics v27 (IBM Corp., Armonk, NY, USA).

Given the exploratory nature of the study and the lack of prior data to estimate effect size, no formal a priori sample size calculation was performed. Continuous variables are presented as mean ± standard deviation (SD), and categorical variables as absolute frequency and percentage. Group comparisons were performed using Student’s *t*-test or Pearson’s χ^2^ test, as appropriate. Biomarker levels were compared using one-way ANOVA and linear regression models adjusted for GA at amniocentesis. To account for other potential confounding factors, multivariable linear regression analyses were performed based on clinical relevance baseline variables expected to influence the oxidative stress, including smoking status, diabetes, maternal age and body mass index (BMI).

The distribution of 8-iso-PGF_2_α concentrations was assessed for normality. Given the expected right-skewed distribution of biomarker data, log-transformation was explored. Sensitivity analyses excluding extreme values (defined as values beyond 1.5 times the interquartile range) were performed to evaluate the robustness of the findings. A *p*-value < 0.05 was considered statistically significant.

## 3. Results

A total of 123 fetuses were included in the study, comprising 83 CHD and 40 controls. Baseline characteristics were similar between the groups (Table 1), including maternal age, rates of hypothyroidism, pre-gestational diabetes, and preeclampsia, except for smoking habit, which was more frequent in the control group (*p* < 0.05). GA at amniocentesis and at third-trimester ultrasound did not differ between groups, and fetal growth and Doppler parameters were comparable at both time points (Table 2). Pregnancy complications and perinatal outcomes including GA at delivery, type of delivery and birthweight were also similar. Within the CHD group, postnatal data confirmed a representative spectrum of cardiac defects, with proportions of critical neonatal and univentricular CHD consistent with expected epidemiologic distributions.

**Figure 1:**
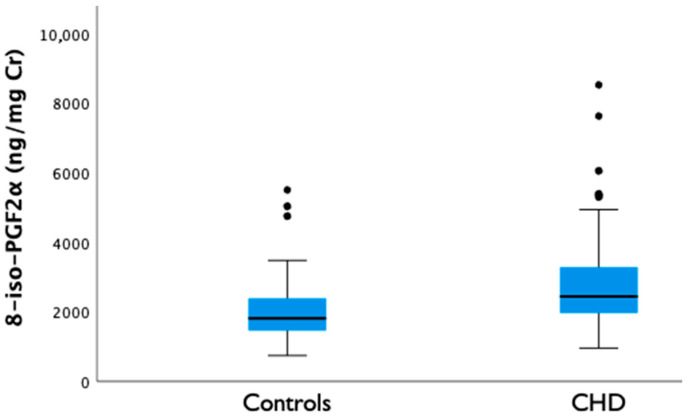
Amniotic fluid 8-iso-PGF_2_α concentrations in fetuses with congenital heart defects and controls. Data are expressed as mean. CHD, congenital heart defect.

CHD fetuses exhibited higher OS biomarkers in AF compared with controls, as evidenced by the significantly elevated 8-iso-PGF_2_α concentration (2849 ± 1377 vs. 2088 ± 1087 ng/mg Cr, *p* ≤ 0.001; mean difference 761 ng/mg Cr (95% CI 270–1253) (Figure 1) after the multivariable linear regression model. Results were robust to log-transformation of 8-iso-PGF_2_α concentrations and to the exclusion of outliers in sensitivity analyses. A few upper-tail outliers were observed in both groups. Among CHD fetuses, these included one case of truncus arteriosus type I, one fetus with a hypoplastic aortic arch, two cases of d-transposition of the great arteries and two large perimembranous ventricular septal defects. In the control group, outliers consisted of one right aortic arch and two aberrant right subclavian arteries. Aside from the specific lesion, the outlier cases did not share distinctive baseline characteristics. Notably, four CHD cases with outlying values underwent amniocentesis at a later GA (>36 weeks) so the analyses were adjusted by GA as detailed in *Methods*.

When analyzed across the predefined study subgroups based on anatomical type, expected placental contribution to brain perfusion, and fetal and postnatal parameters, amniotic 8-iso-PGF_2_α levels did not differ between groups (Table 3). Thus, no significant differences were detected according to the type of CHD or any of the stratification frameworks applied, although a non-significant trend toward higher levels was noted in fetuses with CHD associated with reversed flow in the ductus arteriosus (*p* = 0.06). These subgroup analyses were exploratory and hypothesis-generating; accordingly, no adjustment for multiple comparisons was performed.

Additional exploratory analyses showed no significant correlations between amniotic fluid 8-iso-PGF_2_α concentrations and fetal Doppler indices, including z-scores of the umbilical artery, middle cerebral artery, ductus venosus, or cerebroplacental ratio, nor with head circumference (HC) z-scores. Stratification of cases according to different percentiles of 8-iso-PGF_2_α concentrations did not reveal any clustering of specific congenital heart disease subtypes across percentile categories. Furthermore, subgroup analyses comparing pregnancies complicated by maternal diabetes versus non-diabetic pregnancies, as well as preeclampsia versus pregnancies without preeclampsia, showed no significant differences in AF 8-iso-PGF_2_α levels (mean 2117.73 vs. 2622.82 ng/mg Cr, *p* = 0.154 and mean 3386.61 vs. 2561.12 ng/mg Cr, *p* = 0.533, respectively), and these findings should be interpreted with caution given the limited number of cases with maternal diabetes or preeclampsia in the study population.

## 4. Discussion

Our results provide the first evidence that fetuses with CHD exhibit significantly higher AF concentrations of 8-iso-PGF_2_α compared with controls, indicating increased intrauterine OS. No clear differences were identified across anatomical, expected brain placental perfusion or clinical CHD subgroups, although a trend toward higher levels was noted in fetuses with CHD associated with reversed ductus arteriosus flow. These findings suggest that oxidative imbalance is already established prenatally in CHD.

Our findings are consistent with postnatal studies demonstrating increased OS in neonates with CHD, particularly in cyanotic forms or in those requiring early surgical intervention [2,7,15,16]. Increased urinary and serum 8-iso-PGF_2_α concentrations have been associated with prolonged mechanical ventilation, longer intensive care unit stay, and neurological complications, while delayed postoperative biomarker clearance predicts poorer outcomes [15]. Importantly, our prenatal results indicate that this oxidative imbalance is already present in utero, prior to postnatal circulatory transition or surgical stress. In a previous retrospective case–control study conducted by our group [19], increased o-tyrosine, a marker of protein oxidation, was present in fetuses with CHD, particularly in those with expected lower cerebral oxygenation. However, o-tyrosine provides a more limited view of global OS as it only reflects specific oxidation of amino acids and may be influenced by precursor availability. In contrast, the present study extends this previous work by evaluating 8-iso-PGF_2_α in a larger prospective cohort and by focusing on a widely accepted and stable end-product of non-enzymatic lipid peroxidation, allowing a more robust assessment of OS status.

Although different CHD phenotypes are associated with distinct fetal circulatory patterns and varying degrees of hypoxia, which could theoretically lead to heterogeneous oxidative responses [34,35,36], stratified analyses based on anatomical, brain placental perfusion, hemodynamic, and clinical classifications did not reveal significant subgroup differences. Differing from our previous study, a non-significant trend toward higher 8-iso-PGF_2_α levels was observed only in CHD with reversed ductus arteriosus flow, a marker of ductus-dependent circulation associated with postnatal cyanosis. Overall, these findings suggest that OS may represent a shared pathophysiological feature across the CHD spectrum during fetal life, potentially modulated by feto-placental compensatory mechanisms and intrauterine antioxidant capacity [14,28,37,38]. Although limited subgroup sample sizes may have reduced statistical power, the trend observed in cases with reversed ductal flow highlights the possible influence of fetal hemodynamics and supports further investigation into phenotype-specific vulnerability to OS.

To further contextualize these findings, we examined whether the observed increase in OS could be explained by maternal or obstetric factors known to influence oxidative balance in prenatal conditions other than CHD [39]. Rates of diabetes and preeclampsia were comparable between groups, and although smoking exposure, known to increase OS, was more frequent in the control group, fetal growth parameters and Doppler indices were similar across cases and controls. Collectively, these observations reduce the likelihood that obstetric confounders account for the higher AF 8-iso-PGF_2_α levels observed in fetuses with CHD. Because physiological increases in OS with advancing gestation has been well documented [38,40], cases and controls were matched for GA at sampling, and all analyses were further adjusted for this variable to minimize residual confounding. The persistence of the observed group difference after adjustment supports the presence of an intrinsic oxidative burden associated with CHD rather than a GA-related effect.

Mechanistically, redox imbalance in CHD may result from altered fetal hemodynamics, tissue hypoxia, and impaired antioxidant defenses [13]. Disruption in fetal and placental circulations has been linked to hypoxia–inflammation coupling and increased reactive oxygen species generation [2,36,41,42], providing a plausible biological substrate for intrauterine OS. Beyond CHD, recent evidence indicates that OS is a shared mechanism across pregnancy complications characterized by placental dysfunction, hypoxia, and inflammation, including preeclampsia, fetal growth restriction, gestational diabetes, prematurity, and premature rupture of membranes [14,20,21,23,24,27,39,43,44,45,46,47,48,49,50]. In preeclampsia, elevated 8-iso-PGF_2_α correlates with blood pressure, inflammatory cytokines, angiogenic imbalance, and disease severity, supporting a direct link with placental pathology [51,52,53,54,55]. Similar patterns have been described in fetal growth restriction, gestational diabetes, and prematurity [45,46,47,48,49,50,56,57,58,59,60].

Within this context, the finding of increased 8-iso-PGF_2_α in fetuses with CHD extends the relevance of lipid peroxidation beyond placental disorders to conditions primarily defined by fetal cardiovascular maldevelopment. Many forms of CHD are associated with chronic alterations in fetal oxygen delivery, placental structure, and inflammatory signaling, likely converging on oxidative pathways similar to those described in placental-related complications [56,61]. The convergence of clinical and experimental evidence further suggests that OS is not merely a downstream consequence but may actively contribute to abnormal cardiac development. Experimental murine models show that pro-oxidant exposure during organogenesis induces structural cardiac defects, whereas antioxidant interventions mitigate these effects [62], supporting the notion that a proportion of the oxidative burden observed in CHD is environmentally biologically relevant and potentially modifiable [18,63,64].

Although neurodevelopmental outcomes were not assessed in this cohort, previous evidence suggests that OS may contribute to neurological vulnerability in CHD [16,64]. As AF reflects fetal metabolic turnover, elevated 8-iso-PGF_2_α may also provide indirect insight into fetal oxidative exposure, including potential cerebral involvement, supporting its potential value for future risk assessment [64].

This study has several limitations. First, 8-iso-PGF_2_α concentrations were measured using an ELISA-based method. While liquid chromatography–tandem mass spectrometry (LC–MS/M) provides higher analytical specificity, ELISA has been reported as a robust, cost-effective, and widely used approach in clinical research [65]. Moreover, previous studies have demonstrated good correlation between ELISA and mass spectrometry methods for OS biomarker quantification in biological samples [66].

Another limitation could be the assessment of a single OS biomarker. Although 8-iso-PGF_2_α is a well-established and reliable marker of lipid peroxidation, it reflects only one aspect of the redox balance. The absence of complementary oxidative and inflammatory markers limits a more comprehensive characterization of the redox environment. Nevertheless, the use of 8-iso-PGF_2_α provides a focused and biologically relevant assessment of lipid peroxidation. Future studies incorporating broader biomarker panels are warranted.

Although larger than previous prenatal works, the sample size was limited, and subgroup analyses should therefore be interpreted with caution. Only a single 8-iso-PGF_2_α measurement per fetus was available. The observational nature of the study precludes establishing a causal relationship, and residual confounding related to individual variability in placental function or genetic susceptibility to oxidative imbalance cannot be completely excluded [5,14]. In addition, a small number of control fetuses presented minor structural anomalies that were not expected to influence OS or AF composition.

Future research should include multicenter longitudinal studies to characterize AF 8-iso-PGF_2_α dynamics across gestation, investigate redox-based phenotypic subclassifications, and examine associations with neurodevelopmental and perioperative outcomes. Standardized LC-MS/MS assays, assessment of ratio metrics (8-iso-PGF_2_α/PGF_2_α), and harmonized sampling windows will be essential to define clinical utility. In addition, whether maternal nutritional, lifestyle, or pharmacologic interventions can modulate fetal oxidative status and improve outcomes in this high-risk population remains to be determined.

Despite these limitations, this study provides the first intrauterine evidence of increased OS in CHD using AF 8-iso-PGF_2_α. By analyzing AF, the study captures the fetal metabolic environment prior to birth-related circulatory and inflammatory transitions, avoiding major postnatal confounders such as reoxygenation and surgical stress [7,15,16]. Careful cohort selection, exclusion of congenital extracardiac and genetic anomalies, balanced maternal and fetal characteristics between groups, and multivariable adjustment strengthen the robustness of the findings. Importantly, sensitivity analyses excluding outliers and analyses using log-transformed data yielded consistent results, supporting the stability of our results.

Overall, these findings provide novel intrauterine evidence of OS in CHD, supporting its role as an early feature of the disease while warranting further investigation into its mechanistic and clinical implications.

## 5. Conclusions

This study provides novel intrauterine evidence of increased AF 8-iso-PGF_2_α concentrations in fetuses with CHD compared with controls. These differences persisted after adjustment for GA and potential maternal and gestational confounders, supporting an association between CHD and increased intrauterine OS. While 8-iso-PGF_2_α may represent a promising biomarker of fetal oxidative burden, these findings should be interpreted with caution given their exploratory and observational nature. Further multicenter longitudinal studies are needed to confirm these findings and to better define the potential clinical relevance of this biomarker, including its relationship with prenatal brain development, postnatal neurodevelopment, and perioperative outcomes.

## Figures and Tables

**Table 1:** Baseline, gestational and perinatal characteristics of the study groups.

	Controls (*n* = 40)	Cases (*n* = 83)
**Maternal data**		
Age (years)	33 ± 6	33 ± 5
Smoking (%)	10.5	1.2 *
Nulliparity (%)	41	55
BMI	31 ± 7	27 ± 5
Caucasian ethnicity (%)	80	71
Hypothyroidism (%)	0	6
Pre-gestational diabetes (%)	3	5
**Gestational data**		
Gestational diabetes (%)	11	7
Preeclampsia (%)	5	1
GA at amniocentesis (weeks)	25 ± 1	27 ± 6
GA at 3rd trimester US (weeks)	33 ± 2	32 ± 8
**Perinatal data**		
GA at birth (weeks)	39 ± 12	39 ± 2
Birth weight (g)	3258 ± 405	3138 ± 525
Birth weight centile	53 ± 17	43 ± 29
Labor induction (%)	44	49
Cesarian section (%)	39	27
Female (%)	44	41
Arterial pH	7.21 ± 0.08	7.25 ± 0.06 *
5 min. Apgar score < 7 (%)	0	2.4
**Amniocentesis indications in the control group**		
Minor cardiac defects *: 16 (40%) Unconfirmed CHD postnatal **: 5 (12.5%)		
Abnormally positioned feet: 10 (25%)		
High-risk aneuploidy screening with normal genetic result: 7 (17.5%)		
Hepatic echogenic focus: 2 (5%)		

Data are expressed as mean ± SD or %. * *p*-value < 0.05 calculated by *t*-test or Chi-square as appropriate. GA, gestational age; BMI, body mass index; US, ultrasound. * Minor cardiac defects included 9 cases of aberrant right subclavian artery, 6 cases of right aortic arch and 1 fetus with a small muscular ventricular septal defect. ** Non-confirmed fetal CHD included 2 cases of small perimembranous ventricular septal defect suspected, 1 case of apical muscular ventricular septal defect, 1 fetus with high risk of coarctation of the aorta and 1 case with mild aortic valvular stenosis.

**Table 2:** Fetal growth and feto-maternal Doppler characteristics in fetuses with congenital heart defects.

Feto-Placental Characteristics at Amniocentesis	CHD (*n* = 83)
GA (weeks)	25 ± 5
EFW (g)	1261 ± 884
EFW centile	47 ± 31
Middle cerebral artery PI (z-score)	−0.2 ± 0.9
Umbilical artery PI (z-score)	+0.4 ± 1.5
Cerebroplacental ratio (z-score)	+0.4 ± 1.1
Ductus venosus PI (z-score)	+0.1 ± 1.8
Mean uterine artery (z-score)	+0.2 ± 1.0
**Feto-Placental Characteristics** **at Third Trimester**	
GA (weeks)	33 ± 4
Estimated fetal weight (g)	2086 ± 494
EFW centile	44 ± 29
Middle cerebral artery PI (z-score)	+0.1 ± 1.2
Umbilical artery PI (z-score)	−0.3 ± 1.1
Cerebroplacental ratio (z-score)	+0.3 ± 1.4
Ductus venosus Doppler PI (z-score)	−0.1 ± 1.8
Mean uterine artery PI (z-score)	+0.1 ± 1.0

Data are expressed as mean ± SD. CHD, congenital heart defect. GA, gestational age. EFW, estimated fetal weight; PI, pulsatility index.

**Table 3:** 8-iso-PGF_2_α levels across congenital heart defect subgroups.

CHD Classification (*n* = 83)	Stratification	GA at Amniocentesis (Weeks)	8-Iso-PGF_2_α (ng/mg Cr)	*p*-Value
**Anatomical classification**	Right CHD (*n* = 10)	26 ± 6	3129.37 ± 1315.60	0.64
	Left CHD (*n* = 11)	27 ± 5	2300.01 ± 911.33	
	Septal CHD (*n* = 22)	29 ± 6	2770.96 ± 1585.21	
	Conotruncal CHD (*n* = 39)	25 ± 6	2973.98 ± 1395.82	
	Other types (*n* = 1)	25	2925.60	
Expected brain placental perfusion classification *	Low placental contribution (*n* = 28)	27 ± 6	2964.38 ± 1171.47	0.70
	Intermediate placental contribution (*n* = 36)	27 ± 6	2887.68 ± 1651.30	
	Near-to-normal placental perfusion (*n* = 15)	27 ± 5	2543.15 ± 1020.64	
**Obstructed aortic flow**	Yes (*n* = 16)	28 ± 6	2550.60 ± 832.00	0.14
	No (*n* = 67)	26 ± 6	2920.24 ± 1473.97	
**Reversed aortic isthmus flow**	Yes (*n* = 10)	29 ± 6	2816.09 ± 1028.31	0.28
	No (*n* = 73)	26 ± 6	2853.49 ± 1424.05	
**Reversed ductus arteriosus flow**	Yes (*n* = 6)	26 ± 6	3663.23 ± 802.20	0.06
	No (*n* = 77)	27 ± 6	2785.54 ± 1395.64	
**Intracardiac septal defect**	Yes (*n* = 48)	27 ± 6	2877.03 ± 1487.61	0.88
	No (*n* = 35)	26 ± 5	2810.52 ± 1230.01	
**Critical neonatal CHD**	Yes (*n* = 44)	27 ± 6	2682.85 ± 1382.81	0.11
	No (*n* = 39)	27 ± 6	2996.24 ± 1371.19	
**Univentricular CHD**	Yes (*n* = 5)	26 ± 3	2589.77 ± 765.78	0.70
	No (*n* = 78)	27 ± 6	2865.60 ± 1408.86	

Data are expressed as mean ± SD. *p*-value calculated by linear regression adjusted by gestational age (GA) at amniocentesis. CHD, congenital heart defect. * The low placental contribution group was predominantly composed of 23 d-transpositions of the great arteries and related variants, 2 coarctation of the aorta with reversed aortic isthmus flow, 1 critical aortic stenosis and 4 other complex congenital heart defects. The intermediate placental contribution group comprised 18 ventricular septal defects, 11 tetralogies of Fallot and 8 other mixed-perfusion anomalies. The near-normal placental contribution group included 2 coarctation of the aorta without reversed isthmic flow, 1 congenitally corrected transposition of the great arteries without associated defects, 1 double outlet right ventricle and 11 other right-sided cardiac anomalies.

## Data Availability

The original contributions presented in this study are included in the article. Further inquiries can be directed to the corresponding author.

## Abbreviations

The following abbreviations are used in this manuscript:

AF	Amniotic fluid
CHD	Congenital heart disease
GA	Gestational age
HC	Head circumference
OS	Oxidative stress
8-iso-PGF_2_α	8-iso-prostaglandin F_2_α
o-Tyr	ortho-tyrosine
Cr	Creatinine
ELISA	Enzyme-linked immunosorbent assay
LC-MS/MS	Liquid chromatography–tandem mass spectrometry
PI	Pulsatility index
SD	Standard deviation
CI	Confidence interval
ANOVA	Analysis of variance
ng/mg	nanograms per milligram
